# The Relationship between Robot’s Nonverbal Behaviour and Human’s Likability Based on Human’s Personality

**DOI:** 10.1038/s41598-018-25314-x

**Published:** 2018-05-30

**Authors:** Chidchanok Thepsoonthorn, Ken-ichiro Ogawa, Yoshihiro Miyake

**Affiliations:** 1 Department of Computational Intelligence and Systems Science, Tokyo Institute of Technology, Yokohama, Japan; 2 Department of Computer Science, Tokyo Institute of Technology, Yokohama, Japan

## Abstract

At current state, although robotics technology has been immensely developed, the uncertainty to completely engage in human-robot interaction is still growing among people. Many current studies then started to concern about human factors that might influence human’s likability like human’s personality, and found that compatibility between human’s and robot’s personality (expressions of personality characteristics) can enhance human’s likability. However, it is still unclear whether specific means and strategy of robot’s nonverbal behaviours enhances likability from human with different personality traits and whether there is a relationship between robot’s nonverbal behaviours and human’s likability based on human’s personality. In this study, we investigated and focused on the interaction via gaze and head nodding behaviours (mutual gaze convergence and head nodding synchrony) between introvert/extravert participants and robot in two communication strategies (Backchanneling and Turn-taking). Our findings reveal that the introvert participants are positively affected by backchanneling in robot’s head nodding behaviour, which results in substantial head nodding synchrony whereas the extravert participants are positively influenced by turn-taking in gaze behaviour, which leads to significant mutual gaze convergence. This study demonstrates that there is a relationship between robot’s nonverbal behaviour and human’s likability based on human’s personality.

## Introduction

Robotics technology, especially social robots, interactive robots, or collaborative robots, has been immensely developed as a part of our daily life. It has been integrated in our living environments, for example, in school^1–3^, in museum^4,5^, in hospital^6–8^, or even in our household^9–11^. Although the developments of robotics technology have been remarkably improved, the uncertainty to completely engage in the interactions with robots is still broadly arisen among users or humans due to their concerns on safety^12^, eeriness^13^, and non-human-likeness in robots^14^, which cause negative attitudes toward robots^15–17^. Many robotics and human-robot interaction studies then started to concern not only on developing the more human-likeness in robots but also investigating the influence of human factors as well in order to minimize negative feelings toward robots and find approaches to increase positive feelings or likability toward robots.

Human’s personality is one of the human factors that is widely investigated, especially in introversion-extraversion dimension. The famous study of Jung defined the introverts as conservative, mysterious, and shy and that of extraverts as outgoing and sociable^18^. Eysenck also proposed that introverts’ higher arousability leads them to avoid from external stimulation and that extraverts’ low arousability leads them to seek for external stimulation^19^. Many previous studies have been examined communication style (both verbal and nonverbal), and investigated similarities and differences between introverts and extraverts in order to enhance the effectiveness and attractiveness in communication^20,21^. They found that personality preferences differ in expressed behaviours and traits, which can be implied as individual’s likability toward the interactional partner. In human-human interaction, the previous studies revealed that personality compatibility can enhance, for instance, interaction quality in doctor-patient relationship^22^ and satisfaction in service employee-customer relationship^23^. Furthermore, for collaborative works, the previous study also suggested that the compatibility in coworkers’ personality can result in a smooth and effective collaboration^24^. These previous studies in human-human interaction provided evidences suggesting that human’s personality should be taken into the account for investigation in human-robot interaction as well in order to increase likability toward robots. Many studies in human-robot interaction then started to concern the influence of human’s personality. The previous studies in human-robot interaction also demonstrated that personality compatibility between human and robot via both verbal and nonverbal behaviours can enhance human’s likability toward robots. In Nass’s and Aly’s studies, they both showed that introvert participants prefer the interaction with introvert characteristics voices while the extravert participants prefer extravert characteristics more^25,26^. Moreover, some studies demonstrated that people prefer the interaction with an assistive robot whose personality matches their own, which results in higher satisfaction and better task performance^27,28^. These previous studies evinced that the robot’s behaviours that can express the characteristics of its personality and are compatible with human’s personality can facilitate higher likability toward the robot. Furthermore, previous studies also found that there is a relationship between personality and bodily communication in human-human interaction and it is evidenced in their nonverbal behaviours^29,30^. These previous studies provided evidences showing that nonverbal behaviour compatibility is surely a subpart of personality compatibility and these compatibilities help facilitating communication in human-human interaction. However, for human-robot interaction, it is still unclear whether there is nonverbal behaviour compatibility between human and robot or not and it leaves following unanswered questions. Firstly, does specific means and strategy of robot’s nonverbal behaviour influences and enhances likability from human with different personality traits (Introvert and Extravert)? Secondly, is there any relationship between robot’s nonverbal behaviour and human’s likability based on human’s personality? We hypothesized that nonverbal communication style preference in human-robot interaction differs between personality traits.

In this study, we then aim to investigate whether specific means (gaze or head nodding behaviour) and strategy (Backchanneling or Turn-taking) of robot’s nonverbal behaviour influences and enhances likability in human with specific personality (Introvert and Extravert). In other words, is there any relationship between robot’s nonverbal behaviour and human’s likability based on human’s personality and whether it is really expressed through their interactional behaviours with the robot accordingly. For our study, we selected gaze and head nodding behaviours (mutual gaze convergence and head nodding synchrony) as the main focuses in this investigation as these nonverbal behaviours are claimed to be effective means to indicate the success and involvement with interactional partner^31,32^. Also, gaze and head nodding behaviours are considered as nonverbal cues for both backchanneling^33^ and turn-taking in communication^34^. Furthermore, gaze behaviour is asserted to be means for expressing not only attention and interest^35^ but also positive feelings like affection^36^ and many other social information as well^37,38^. Head nodding behaviour is also asserted to be one of obvious and prominent nonverbal behaviours that the interactional partner can easily perceive, representing attentiveness, understanding and acceptance to the interactional partner^39,40^. Furthermore, many previous studies also supported that mutual gaze convergence between 2 individuals can represent quality and satisfaction in the interaction^41^ and positive feelings to each other^35^ while head nodding synchrony between 2 individuals can indicate satisfaction increment^42,43^ and positive attitude stimulation^44^.

**Figure 1:**
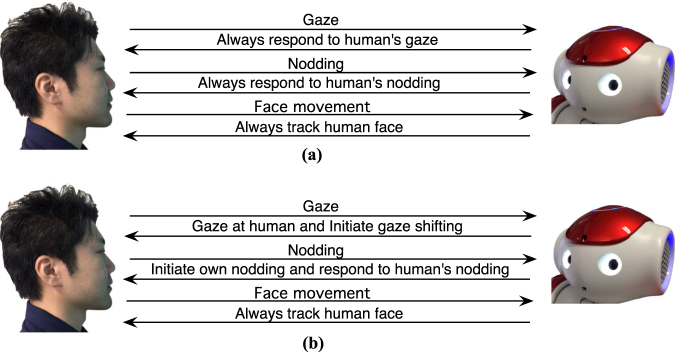
Robot’s behaviour strategies. (**a**) Backchanneling strategy’s behaviour scenario between human and robot. (**b**) Turn-taking strategy’s behaviour scenario between human and robot.

To explore the relationship between robot’s nonverbal behaviour and human likability based on human’s personality in human-robot interaction, we developed 2 robot’s behaviour strategies for NAO robot: Backchanneling strategy and Turn-taking strategy, focusing on robot’s gaze and head nodding behaviours. Speaking with gestures is a common behaviour in all 2 strategies. With Backchanneling strategy, the robot will always give responses via its gaze and head nodding as backchannelings^33^ to human. When human initiates head nodding, the robot will nod as a response. When human makes head movement, the robot will move its head accordingly and always gaze back to the human (Fig. 1(a)). This strategy is similar to master-slave concept^45^, which is typically used in general robots where the robot takes slave role and follows its human master during the interaction. For Turn-taking strategy, turn-taking concept of human-human interaction is applied. In human-human interaction, humans do turn taking of being leader and follower with the interactional partner from time to time^34,46,47^. In gaze behaviour, humans do not always gaze at the interactional partner. They both make and break eye contact and take turns whether who joins or averts from mutual gaze convergence^48,49^. In case of head nodding behaviour, turn-taking is also occurred in human-human interaction. Humans both take a lead in initiating head nodding^40^ and follow or respond to their interactional partner’s head nodding from time to time^33^. With Turn-taking strategy, the robot will not always look at its interactional partner. It will break gaze by doing gaze shifting from time to time when too long eye contact with human is occurred. Also, it will perform turn-taking on its head nodding behaviour similar to human-human interaction (Fig. 1(b)).

In this study, we conducted an experiment on face-to-face, human-robot interaction. The NAO robot took speaker role while the participants took listener role. Before beginning the experiment, the participants were asked to fill in the pre-interaction questionnaire about their demographic and do self-evaluation via online personality test based on Myers-Briggs Type Indicator: MBTI from Myers-Briggs’s studies focusing on introversion and extroversion in order to categorize the participants into Introvert or Extravert group^50,51^. In the experiment, all participants interacted with NAO robot with 2 behaviour strategies (randomly and anonymously), 2 trials in total. The duration of the interaction for each trial is about 2 minutes. In every trial, the robot conveyed the biography of Paul Cezanne, an artist of impressionism art, with gestures to the participants while its gaze and head nodding behaviours were altered according to each behaviour strategy. During the interaction, we recorded the video from the robot’s camera, located on its forehead, and the video from web camera to capture gaze and head nodding behaviours of both human and robot. After the interaction, the participants were asked to answer a post-interaction questionnaire about their preference on robot’s behaviour strategy (Backchanneling or Turn-taking) and their preference on robot’s nonverbal behaviour that can enhance their likability toward the robot the most (gaze or head nodding behaviour). Apart from paper-based measurement, we also measured mutual gaze convergence and head nodding synchrony between human and robot by conducting offline recorded videos analysis as behavioural measurements as well.

## Results

Thirty participants’ questionnaire answers and recorded videos were used for analyses in this study. The participants were categorized into 2 groups based on their personality: 15 introverts and 15 extraverts^26,27^. In this section, we compared the participants’ preference on robot’s behaviour strategy, preference on robot’s nonverbal behaviour that can enhance their likability toward the robot the most, and their interactional behaviours via mutual gaze convergence and head nodding synchrony with the robot in each behaviour strategy as behavioural measurements. Here, we demonstrated the analysis of Introvert and Extravert group, respectively.

### Results of Introvert group’s questionnaire answers

**Figure 2:**
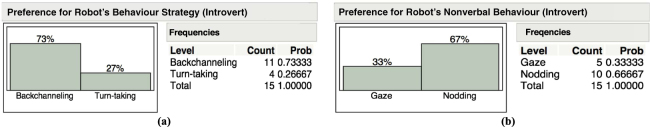
Questionnaire results of Introvert group (n = 15). (**a**) Introvert group’s preference on robot’s behaviour strategy. (**b**) Preference on robot’s nonverbal behaviour.

In Introvert group (n = 15), according to the questionnaire answers, the participants in this group highly prefer Backchanneling strategy (73%, n = 11) to Turn-taking strategy (27%, n = 4). See Fig. 2(a). It is supported by Likelihood ratio test indicating that the preference of Introvert group in Backchanneling strategy is significantly higher than in Turn-taking strategy, χ^2^(1) = 14.16, p = 0.0002. For their preference in robot’s nonverbal behaviour, the Introvert group highly prefers robot’s head nodding behaviour (67%, n = 10) to robot’s gaze behaviour (33%, n = 5) as shown in Fig. 2(b). Also, the Likelihood ratio test supports that the preference of Introvert group in robot’s head nodding behaviour is significantly higher than in robot’s gaze behaviour, χ^2^(1) = 6.93, p = 0.0085.

### Results of Extravert group’s questionnaire answers

**Figure 3:**
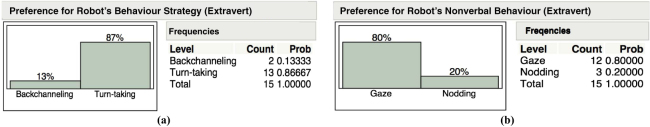
Questionnaire results of Extravert group (n = 15). (**a**) Extravert group’s preference on robot’s behaviour strategy. (**b**) Preference on robot’s nonverbal behaviour.

In Extravert group (n = 15), according to the questionnaire answers, the participants in this group highly prefer Turn-taking strategy (87%, n = 13) to Backchanneling strategy (13%, n = 2). See Fig. 3(a). This is supported by the result of Likelihood ratio test, which indicates that the preference of Extravert group in Turn-taking strategy is significantly higher than in Backchanneling strategy, χ^2^(1) = 41.18, p < 0.0001. For their preference in robot’s nonverbal behaviour, the Extravert group highly prefers robot’s gaze behaviour (80%, n = 12) to robot’s head nodding behaviour (20%, n = 3) as shown in Fig. 3(b). Also, the Likelihood ratio test supports that the preference of Extravert group in robot’s gaze behaviour is significantly higher than in robot’s head nodding behaviour, χ^2^(1) = 24.95, p < 0.0001.

### Results of mutual gaze convergence occurrence percentage

**Figure 4:**
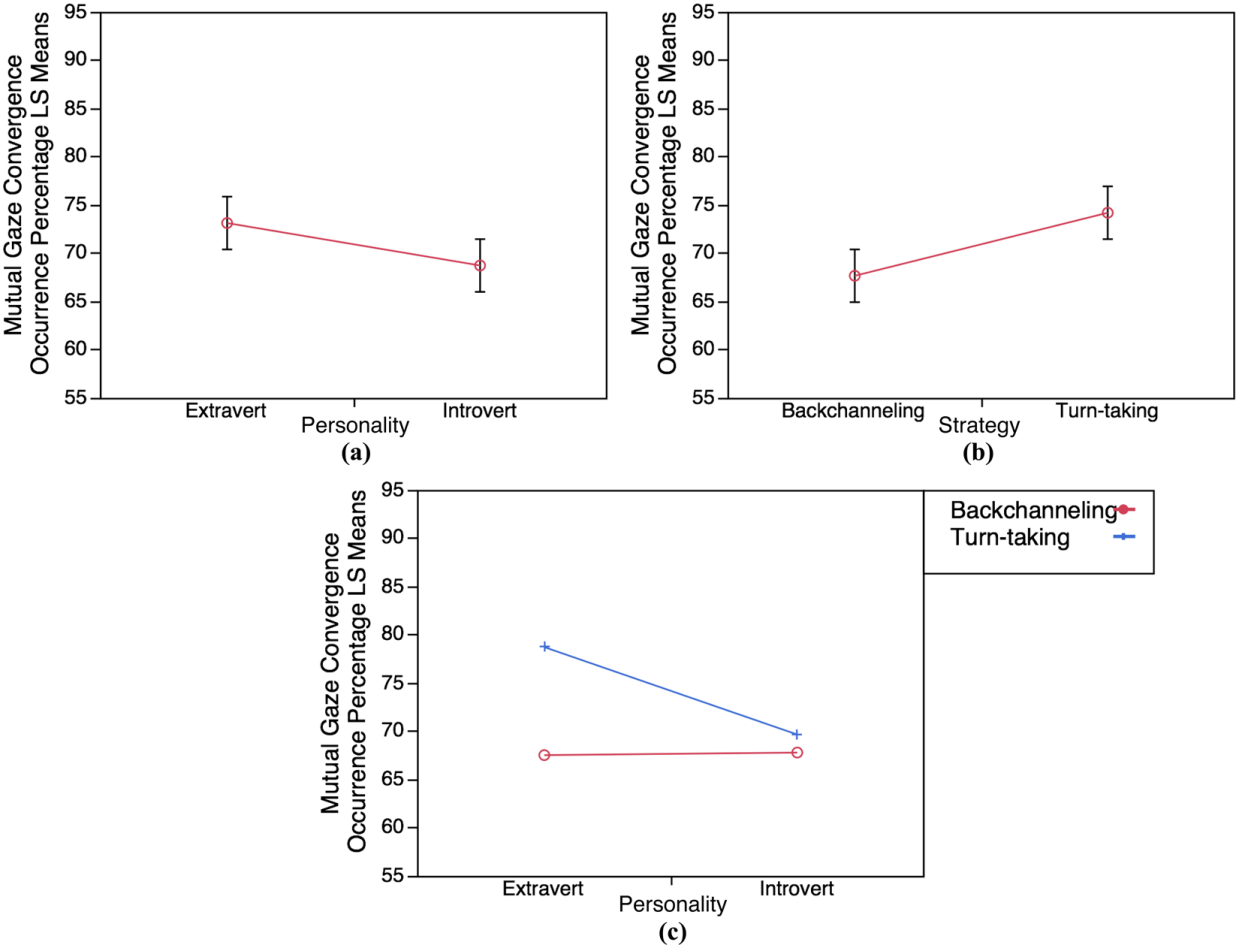
Least Squares Means results of mutual gaze convergence occurrence percentage (LS Means). (**a**) LS Means for personality effect. (**b**) LS Means for strategy effect. (**c**) LS Means for interaction effect (personality × strategy).

We analyzed the mutual gaze convergence occurrence percentage data by conducting a 2 × 2 factorial design: personality (Introvert and Extravert) × strategy (Backchanneling and Turn-taking) using ANOVA. According to the ANOVA result for mutual gaze convergence, we found that both main effects are significant, indicating that the mutual gaze convergence occurrence percentage in Extravert group is significantly higher than in Introvert, F(1, 56) = 11.47, p = 0.0013 (Fig. 4(a)), and that the mutual gaze convergence occurrence percentage is significantly higher when using Turn-taking strategy than Backchanneling strategy, F(1, 56) = 5.2, p = 0.0264 (Fig. 4(b)). Moreover, the interaction effect is also significant, F(1, 56) = 5.85, p = 0.0188, providing an evidence indicating that the effect of strategy depends on the participants’ personality (Extravert personality and Turn-taking strategy), F(1, 56) = 5.85, p = 0.0188 (Fig. 4(c)).

### Results of head nodding synchrony occurrence percentage

**Figure 5:**
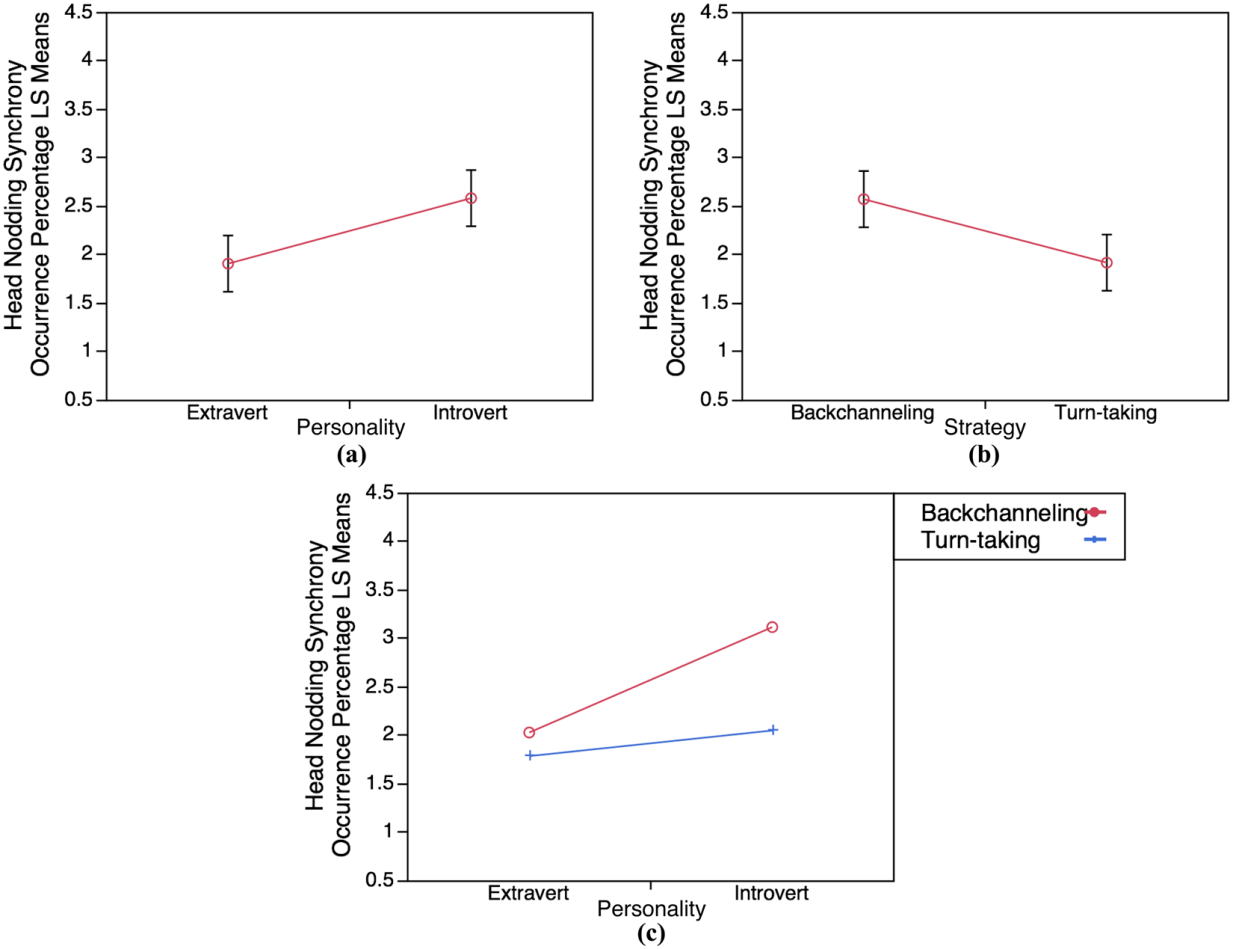
Least Squares Means results of head nodding synchrony occurrence percentage (LS Means). (**a**) LS Means for personality effect. (**b**) LS Means for strategy effect. **(c)** LS Means for interaction effect (personality × strategy).

We also analyzed the head nodding synchrony occurrence percentage data by conducting a 2 × 2 factorial design: personality (Introvert and Extravert) × strategy (Backchanneling and Turn-taking) using ANOVA. We also found that both main effects are significant, indicating that the head nodding synchrony occurrence percentage in Introvert group is significantly higher than in Extravert, F(1, 56) = 10.91, p = 0.0017 (Fig. 5(a)), and that the head nodding synchrony occurrence percentage is significantly higher when using Turn-taking strategy than Backchanneling strategy, F(1, 56) = 10.25, p = 0.0023 (Fig. 5(b)). Furthermore, the interaction effect is also significant, providing an evidence that the effect of strategy depends on the participants’ personality (Introvert personality and Backchanneling strategy), F(1, 56) = 4.05, p = 0.049 (Fig. 5(c)).

## Discussion

To explore the influence factors of the outcomes and investigate the relationship between robot’s nonverbal behaviour and human’s likability based on human’s personality, we categorized the participants into 2 groups in regard to their personality: Introvert and Extravert, and investigated gaze and head nodding behaviours (mutual gaze convergence and head nodding synchrony) between human and robot in 2 interaction strategies (Backchanneling and Turn-taking). The results demonstrate that the Introvert group has significant preference on Backchanneling strategy compared to Turn-taking strategy. The result on their preference in robot’s nonverbal behaviour also indicates that the participants in Introvert group significantly prefer robot’s head nodding behaviour as it can enhance their likability toward the robot more than robot’s gaze behaviour. The results from Introvert group’s preferences are supported by their interactional behaviours with the robot via mutual gaze convergence and head nodding synchrony. The participants in Introvert group expressed significantly higher head nodding synchrony with the robot in Backchanneling strategy than the robot in Turn-taking strategy while no statistical significance is found in mutual gaze convergence. On the other hand, for Extravert group, the results demonstrate that the participants in this group have significant preference on Turn-taking strategy compared to Backchanneling strategy. They also significantly prefer robot’s gaze behaviour as it can enhance their likability toward the robot more than robot’s head nodding behaviour. These preferences of the participants in Extravert group are supported by their interactional behaviour, similar to the Introvert group but in the opposite direction. The participants in Extravert group expressed significantly higher mutual gaze convergence with the robot in Turn-taking strategy than the robot in Backchanneling strategy while no statistical significance is found in Head nodding synchrony. Here, our findings indicate contradictions in participants’ preferences and interactional behaviours between Introvert group and Extravert group.

According to the results from questionnaire and interactional behaviours, it indicates that nonverbal communication style preference in human-robot interaction differs between personality traits. It suggests that the participants in Introvert group give priority to the importance of backchanneling than turn-taking, especially via head nodding behaviour. As we can see from the results, they positively engage and have high likability in the interaction with the robot that can provide head nodding responses as in master-slave concept. In other words, they highly enjoy the interaction where the robot responds to their head noddings as backchanneling, however, only when necessary (only when they nodded) and not too expressive. And it is really expressed and manifested in high occurrence percentage in head nodding synchrony during the interaction. In contrary, the participants in Extravert group give priority to the importance of turn-taking more than backchanneling, particularly via gaze behaviour. As we can see from the results, they have high likability and prefer to interact with the robot that can express its gaze behaviour with Turn-taking strategy where both human and robot can be expressive and take turn on who joins or averts from mutual gaze convergence. It is really established and leads to high occurrence percentage of mutual gaze convergence in the interaction. These findings are consistent with the psychology studies in human-human interaction where they found a relationship between communication behaviours and personality traits (Introversion-Extraversion), which could be implied as individual’s likability or comfort in the interaction^20,52^. They used MBTI framework to examine the relationships between personality and communication style. They found that the introvert participants had higher levels of communication apprehension than extravert participants, which means that the introvert participants seem to express and prefer less interaction elements, for instance, eye contact or expressive communicator style^53,54^. Here, we can infer that the participants in Introvert group are most likely to prefer the interaction with the robot that expresses conservative, not too expressive, and on-command behaviour and provides precise responses to the participants. In this case, robot’s head nodding behaviour with backchanneling strategy may fulfill their preference and their likability toward the robot is really reflected via their occurrence percentage of head nodding synchrony with the robot. In contrary, for the Extravert group, they are most likely to prefer the robot with energetic, active, and expressive behaviour that can communicate interactively with humans. In this case, robot’s gaze behaviour with turn-taking strategy may fulfill their preference and their occurrence percentage of mutual gaze convergence also reflects their likability toward the robot accordingly. Furthermore, the findings in our study also align with similarity-attraction theory^55,56^, show consistency with previous studies on similarity-attraction effect in human-technology interaction^25–28,57,58^, yet, provide an extension of what means and strategy of robot’s nonverbal behaviour that can significantly increase human’s likability regard to human’s personality.

Here, we can infer that there is nonverbal behaviour compatibility between human and robot and it does take part in facilitating likability toward the robot in human-robot interaction. All people do not receive the interaction enjoyment or satisfaction with the robot in the same direction. People with different personality prefer the robot to interact with them differently, especially via specific means and strategy of robot’s nonverbal behaviour, and their likability is also expressed via their interactional behaviour with the robot accordingly. Furthermore, emphasizing the right means and strategy of robot’s nonverbal behaviour and interactional behaviour between human and robot can result in higher likability in human-robot interaction in respect to human’s personality.

In this study, we developed only 2 robot’s behaviour strategies that are differed in gaze and head nodding behaviours. We specified the term “nonverbal behaviour” intending to only refer to gaze and head nodding behaviours of the robot. Therefore, this study is limited and can be referred to as the study on gaze and head nodding behaviours in robot only. Other nonverbal behaviour elements, such as facial expression, proxemics, posture, utterance, etc., should be included for further investigations. Apart from robot’s nonverbal behaviour, the questionnaire should be more precise and include human-likeness, eeriness and affinity aspects to completely evaluate the interaction with the robot. These data could further clarify the differences of participants’ perspective toward each interaction with the robot. Also, more human-robot interactional behaviours, other than mutual gaze convergence and head nodding synchrony, should be observed as behavioural measurements for the future works. Moreover, further study with more participants is required in order to obtain more significant and generalized results.

In conclusion, this study is an exploratory work investigating the relationship between robot’s nonverbal behaviours (gaze and head nodding) and human’s Likability based on human’s personality (Introvert and Extravert) and the influence of robot’s nonverbal behaviour toward human’s likability. Our results offer interdisciplinary findings. In psychology viewpoint, our study demonstrates that a specific group of people is positively affected by specific means and strategy of robot’s behaviour and it is reflected through their interactional behaviour with the robot. The introvert participants are positively affected by Backchanneling strategy of robot’s head nodding behaviour, which results in substantial head nodding synchrony with the robot whereas the extravert participants are positively influenced by Turn-taking strategy in gaze behaviour between themselves and the robot, which leads to significant mutual gaze convergence during the interaction. Here, we discovered that there is a relationship between robot’s nonverbal behaviour and human’s likability based on human’s personality. Also, emphasizing the right means and strategy of robot’s nonverbal behaviour and interactional behaviour between human and robot can result in the higher likability in respect to human’s personality. In robotics viewpoint, our study provides a guideline for designing robot’s behaviour model on what means and strategy that the developers should emphasize in order to fulfill human’s satisfaction and pleasure in human-robot interaction. In other words, pulling the right trigger for the right target group can result in the higher likability. With the integration of studies on both appearance and behaviour aspects in future works, we can further pursue human-likeness in robot whilst gaining more human’s likability in human-robot interaction at the same time.

## Methods

### NAO robot

We employed NAO robot for human-robot interaction in this study. NAO robot is a humanoid robot produced by Aldebaran Robotics (France). NAO robot comprises with sensors, gyroscopes, accelerometer, microphone, speaker, and camera, which embedded on its forehead. Additionally, NAO robot is equipped with a software suite, which allows the developers to fully program and control the NAO robot platform (SDK package with NAOqi API).

### Robot’s behaviours

We programmed 5 main robot’s behaviours, which are face tracking, gaze shifting, nodding initiating, nodding responding, and speaking with gestures, using Python language.

#### Face tracking

With face tracking behaviour, the robot performs human face detection using its camera and keeps tracking the detected human face. The detected human face is always located at the middle of the frame in the robot’s vision (via camera). To enable face tracking behaviour in NAO robot, we applied ALFaceDetection and ALFaceTracker modules from NAOqi.

#### Gaze shifting

In natural human’s nonverbal behaviour in face-to-face interaction, the interactional partners both make and break gaze by averting gaze to elsewhere with each other during the interaction^48,49^. In case of NAO robot, it, unfortunately, has no eyeballs. It cannot avert eyeballs to perform gaze breaking from the interactional partner similar to human. To allow the robot to perform such human-like behaviour, we alternatively programmed gaze shifting behaviour for the robot. With this behaviour, the robot will tilt its head to the left or right (randomly) for 1 degree and tilt back to original position in 1 second. We selected this degree and interval of gaze shifting because in our pilot study, the participants reported that it is perceived as gaze breaking enough while it is not too eerie to distract the participants during the interaction, which is a good choice to be considered as a substitution of eyeballs averting in our study. This behaviour will be activated when the robot can detect human face at the middle of its camera vision frame continuously for more than 3, 5, 7, or 9 seconds (randomly) to avert its gaze direction from interactional partner according to our previous studies^59,60^, which suggest that human has momentary mutual gaze convergence or fixation at their partner both in face-to-face communication and collaborative task.

#### Nodding initiating

According to human’s head nodding behaviour, humans do not only unconsciously have head nodding synchrony with their interactional partner^40,59,61,62^, but also initiates own nodding^38^ and responds to interactional partner’s head nodding as backchanneling behaviour as well^33^. We then applied such head nodding behaviour to NAO Robot. We programmed the robot to initiate its own nodding with different frequencies of nodding (randomly), similar to human’s nodding initiating behaviour, when no head nodding is occurred continuously for more than 6, 8, 10, or 12 seconds (randomly).

#### Nodding responding

In case of head nodding responding behaviour, we applied “ALFaceDetection” module from NAOqi to obtain the detected human face center position as reference point. We stored the reference points of the first 90 frames (first 3 seconds) and extracted the minimum and maximum value as the calibration range in order to detect human nodding from robot’s vision via its camera. The minimum and maximum value of the next 30 frames (1 second) is compared with the calibration range. If it is not in the calibration range, the nodding responding behaviour will be activated. The robot will nod with different frequencies (randomly) as backchanneling respond to the detected human head nodding. After each robot’s head nodding is triggered and expressed, the algorithm will enter calibration state for 1 second for updating the calibration range and then continue to detect human’s head nodding again. Therefore, while the robot is nodding, the algorithm will not trigger another nodding simultaneously. If both robot’s gaze and head nodding behaviours are triggered at the same time, we give a priority to head nodding behaviour since head nodding also breaks eye contact between human and robot as well.

#### Speaking with gestures

In this study, we focus only on gaze and head nodding behaviours. We then applied this behaviour as a common behaviour in all strategies. Therefore, the robot will express the same gestures while speaking in every trial. To allow the robot to speak with gestures similar to human’s behaviour^33^, ALAnimatedSpeech module from NAOqi is used. With this module, we could apply contextual mode and word tagging with specific animation, which maps the content that the robot speaks with related gestures.

### Robot’s behaviour strategies

We developed 2 robot’s behaviour strategies to implement with NAO robot: Backchanneling strategy and Turn-taking strategy, by integrating the combination of the robot’s behaviours aforesaid in Robot’s behaviours section.

#### Backchanneling strategy

Backchanneling strategy is based backchanneling behaviours in human-human interaction^33^. It is also similar to master-slave concept in human-robot interaction where human is the master and robot is human’s slave^45^. The robot will always give response to human’s gaze and head nodding as backchannelings while speaking with gestures. In other words, the robot will always give gaze response to human. When the participant tilts his/her head, the robot will track human face accordingly. When the participant nods, the robot will also nod as a response. All behaviours of the robot will be entirely corresponded to the amount of human’s gaze and head nodding behaviours in each trial. The Backchanneling strategy comprises of face tracking without gaze shifting, nodding responding, and speaking with gestures behaviours. See Fig. 1(a).

#### Turn-taking strategy

Turn-taking strategy applies gaze and head nodding behaviour based on human natural behaviour in human-human interaction. This strategy adopts turn-taking concept, particularly in gaze and head nodding behaviour similar to human-human interaction^37,38^. In human-human interaction, humans do turn taking of being leader and follower with the interactional partner from time to time^46^. In this behaviour strategy, the robot will not always pay full attention or respond to human for all time like it does in Backchanneling strategy. In term of its gaze behaviour, the robot will not always gaze at human. It will break gaze or eye contact with the interactional partner when it faces the human’s face for too long by performing gaze shifting behaviour^47,48^. In term of its head nodding behaviour, the robot will not only respond to human’s head nodding as backchanneling but also initiate its own nodding as well^33,38^. Head nodding initiation will also be performed in order to invoke human’s head nodding and to be in sync with human. In other words, the robot will take both leader and follower role during the interaction. Turn-taking strategy comprises of face tracking with gaze shifting, nodding initiating, nodding responding, and speaking with gestures behaviours. See Fig. 1(b).

### Experimental design

The experiment task is one-way, face-to-face, human-robot interaction. The robot took the role of speaker conveying biography of Paul Cezanne, an artist of impressionism art, to a participant. There are 2 trials in each experiment: Backchanneling strategy and Turn-taking strategy. The duration of the interaction for each trial is about 2 minutes. In each trial, the robot conveyed the same content with gestures to the human participant whereas the robot’s gaze and head nodding behaviours are altered according to each behaviour strategy. The protocols and procedures used in this experiment were approved by the Tokyo Institute of Technology’s Ethical Review Board for Epidemiological Studies. The methods were carried out in accordance with the approved guidelines.

There are 30 international participants participating in this experiment, age ranging from 22 to 35 years old, 18 males and 12 females. We categorized the participants into 2 groups based on their personality using an online personality test. The questions in test are based on Myers-briggs’ studies (Myers-Briggs Type Indicator: MBTI) focusing on only introversion and extroversion^50,51^. The test consists of a series of 2 choices question that force the participants to choose one of two answers that reflect 2 poles (introversion-extraversion), 10 scores for each pole, based on the previous studies^20,21,63^. For example, how big is your circle of friends? [A] less than ten. I prefer quality over quantity. [B] I make friend wherever I go, so I have a lot of good friends. If the participant chose [A], the score in introversion pole is increased by 1 point, and 1 point for extraversion pole if choosing [B]. The score is calculated and converted into introversion and extraversion percentage. If the introversion score is >60%, the participants were categorized into Introvert group. The Extravert group is also categorized using the same criteria^63^. The mean and standard deviation of introversion percentage in Introvert group are 84% and 10.6%, respectively. The mean and standard deviation of extraversion percentage in Extravert group are 81.3% and 9.9%, respectively. In this study, there are 15 introvert participants and 15 extravert participants. The participants’ gender, experience with robots, and age are nearly balance between Introvert and Extravert groups. The details are listed in Table 1. With these distributions, personality is, thus, the main factor for this study. To exclude language difficulty effect since the experiment task is conducted in English, all participants are required to be native English speakers or pass standard English proficiency test (TOEFL PBT > 525, TOEFL iBT > 71, TOEIC > 780, IELTS > 6 or SAT). Informed consent was obtained from all participants before participating the experiment. Also, the informed consent for publication of identifying information/images in an online open-access publication was obtained from the participant whose images are appeared in this manuscript.

**Table 1:** Descriptions of participant factor distributions between Introvert and Extravert groups.

Factors	Introvert Group	Extravert Group
Gender	60% male, 40% female (n = 9:6)	60% male, 40% female (n = 9:6)
Experience with robots	60% experienced with robots, 40% had no experience with robots (n = 9:6)	55.3% experienced with robots, 46.7% had no experience with robots (n = 8:7)
Age (mean)	25.7 years old	27.3 years old

This experiment consists of 3 sessions: practice, interaction, and evaluation session. Before starting the experiment, the experimenter explained the experiment task to the participants and asked them to sign consent form and fill in the pre-experiment questionnaire about their demographic and do personality test via online personality test based on Myers-Briggs’s studies in order to categorize the participants in to Introvert or Extravert group^50,51^. In practice session, all participants had 1 practice trial with the robot. In practice trial, the robot conveyed different content and performed only-gesture behaviour strategy so that it would not give a bias on any investigated behaviour strategies. In interaction session, the participant interacted with the robot for 2 trials, regards to the 2 robot’s behaviour strategy: Backchanneling strategy and Turn-taking strategy. We alternated trial order for each participant in order to eliminate the order effect. The last session is evaluation. In this session, all participants were asked to answer the post-interaction questionnaire.

During the interaction, the participant and the robot faced directly to each other, 90 cm apart. The robot took standing position to perform gestures while the participant took sitting position. To minimize the external factors that might affect the participant’s attention and judgment like robot’s appearance and verbal behaviour, we asked the participant to mainly focus on the robot’s nonverbal behaviours during the interaction. For minimal saliency effects, there were neither experimenters nor movable objects presented in the experiment room during the interaction.

Paper-based questionnaire was used for the evaluation in this experiment. The questionnaire was presented to the participants after the second trial. The questions are firstly; which trial do you prefer more? (Trial 1 or Trial 2, robot’s behaviour strategy) and secondly; what robot’s nonverbal behaviour that you perceive as more human-like and prefer more? (gaze or head nodding behaviour). In addition, we observed and measured mutual gaze convergence and head nodding synchrony between the participant and the robot during the interaction as behavioural measurements as well.

### Mutual gaze convergence detection

For mutual gaze convergence detection, we used one web camera to detect human’s gaze direction and we detected the robot’s gaze direction via the robot’s camera on its forehead. We firstly analyzed the human’s gaze direction using eyeLike project (OpenCV C + + ) as a framework to perform eye detection^64^. We extracted left and right eye regions from the detected face bounding box and located the eyes’ center from the eye regions using image gradients technique. Next, we performed straight gaze detection for each eye center using adaptive threshold in order to marginalize the effects of size difference and head movement of individuals^59^. After receiving the results from straight gaze detection for each eye center, we continued to compare the results of the left and right eye in order to detect looking straight in human’s gaze direction. Though ordinary human eyes have symmetry in line of sight, left and right eyes comparison is required to compensate the accuracy of the straight gaze detection results. If at least one side of the eyes is detected as looking straight, we considered that the human is looking straight in that particular frame. After gaining all frames comparison, we converted the result from each frame to each second using most occurrence approach. In case of robot straight gaze detection, we observed from the recorded video of the interactional human from the robot’s camera. With ALFaceTracker modules from NAOqi in NAO robot, the detected human face will always locate at the middle of the robot’s vision when the robot has straight gaze at human. Therefore, we can easily extract straight gaze of the robot from the recorded video. Once we obtained the straight gaze detection results of both human and robot, we compared the straight gaze results, second by second. If the results indicate that both human and robot have straight gaze direction, we assumed this scenario as mutual gaze convergence, and non-mutual gaze convergence, otherwise.

### Head nodding synchrony detection

With the intention to attach no additional tools on the participants to mimic the genuine interaction in real-life scenario, we decided to detect human face and head nodding via the recorded video from web camera using image processing technique in MATLAB^65,66^ instead. We extracted the y-axis position of detected human face as the reference point of human’s head position from each frame since the head nodding behaviour is occurred in up-down direction. If the reference point (y-axis position of the detected human face) of the current frame exceeds the reference point of the previous frame by ± 5 pixels, difference count increases. In this study, we used 30-frame-per-second web camera. We, therefore, detected human’s head nodding in every 30 frames (each 1 second) and since head nodding cannot be seen in a single frame, we inferred head nodding behaviour regards to the difference count of every 30 frames. If the difference count of each 30 frames is at least 5, we can assume that the head nodding behaviour is occurred. In case of the robot head nodding detection, we recorded data of robot’s head motion in each trial, second by second, starting from 0 to the end of the interaction, and marked the data as nodding when robot’s nodding behaviour is activated. After obtaining the head nodding results of both human and robot, we compared the 2 results. If both results indicate head nodding in particular second, we considered this scenario as head nodding synchrony. However, the perfect head nodding synchrony is very rare in real-life scenario, we also considered head nodding of both human and robot that is occurred in ± 1 second as head nodding synchrony as well.
